# Well-Being and Safety in the Workplace

**DOI:** 10.3390/ijerph19148712

**Published:** 2022-07-18

**Authors:** Michele Teodoro, Federica Giambò

**Affiliations:** Department of Biomedical and Dental Sciences and Morphofunctional Imaging, Occupational Medicine Section, University of Messina, 1 Consolare Valeria Street, 98125 Messina, Italy; federica.giambo@unime.it

The workplace represents a critical and time-consuming exposure setting in which workers are continuously exposed to the heterogeneity of hazards, including physical, biological, chemical, and organizational risk factors. Moreover, personal occupational surroundings, diet, and lifestyle behavior can represent a supplementary source of exposure. The worker should be intended in a broad approach, adopting the standpoint of the occupational physician who oversees the psychophysical wellbeing of workers in the workplace.

Growing literature data report that many environmental and occupational pollutants are linked to several pathologies, such as metabolic diseases, altered immune and neurodevelopment systems, endocrine disruption, reproductive disorders, or cancer.

Recent efforts focus on the mechanisms of oxidative stress and the gut microbiota contributions, highlighting the role of the genetic and epigenetic profiles in terms of susceptibility.

Oxidative stress seems to be involved in mechanisms resulting from exposure to numerous risk factors, such as physical and chemical agents [1,2,3]. This modulation could affect the immune system through perturbation of the cytokine balance.

Seeking for prevention, the identification of early alterations in the immune system could be promoted focusing on epigenetic mechanism—in fact, epigenetic alterations are recognized as triggering events of disease development, including neoplastic transformation [4].

Several studies have shown a higher incidence of pathological stress, anxiety, depression, and post-traumatic disorder symptoms, especially among healthcare professionals [5,6]. Among the consequences of stress, we can include several disorders related to psychological discomfort and organic impairment of the gut as irritable bowel syndrome (IBS), recognized as a combination of irritable bowel and irritable brain in the workplace.

The critical role of the gut microbiota in the pathogenesis of IBS has been increasingly studied, and its compositional alteration has recently been considered a crucial factor in the pathogenesis and pathophysiology of typical syndromes [7,8]. The central hypothesis is that alterations in visceral hypersensitivity, gut immunity, and enteric sensory and motor function can alter the microbiota–gut–brain axis and trigger IBS [9]. In this sense, the gut microbiota (GM) plays a crucial role in GI metabolic, protective, and structural functions; thus, the possible role of dysbiosis in IBS patients has been progressively investigated. Increasing evidence supports the potential of GM and its alteration in influencing human health and disease; moreover, GM manipulation may become an appealing therapeutic target avenue for several conditions [10].

Chronic stress can lead to dysbiosis and increased bacterial wall adhesion. At the same time, the interaction between host and microbiota can modulate the neuro-immuno-endocrine systems, suggesting that stress can lead to alterations in the intestinal microbiota, which plays a crucial role in the pathogenesis of IBS. Stress-related changes in the gut microbiota can help maintain contact between the brain and gut. The coexistence of IBS and psychological distress are frequent, and the prevalence of at least one psychiatric disorder typically ranges from 40% to 60%, and up to 80% has been reported [11]. 

Knowing that prevention represents a primary scope of occupational medicine, this integrated approach could be investigated more effectively to study personalized intervention to reduce the risk of incidence or deterioration of chronic diseases. Future research should reveal the molecular mechanisms involved in developing psychophysical illness. This could be the first step required for implementing prevention and health promotion programs in the workplace.

On these premises, this Special Issue aims to involve an assortment of scientific contributions to reduce the gap in uncovering the interdisciplinary research in these topical areas, including studies focusing on the effects of all the occupational risk factors which may influence physical, mental, and social wellbeing.
